# Prevalence and Risk Factors of Internet Addiction Among Employed Adults in Japan

**DOI:** 10.2188/jea.JE20160185

**Published:** 2018-04-05

**Authors:** Hideki Tsumura, Hideyuki Kanda, Nagisa Sugaya, Satoshi Tsuboi, Kenzo Takahashi

**Affiliations:** 1 Faculty of Medicine, Shimane University, Department of Environmental Medicine and Public Health, Shimane, Japan; 2 Graduate School of Medicine, Yokohama City University, Department of Epidemiology and Public Health, Yokohama, Japan; 3 Department of Epidemiology, Fukushima Medical University, Fukushima, Japan; 4 Teikyo University Graduate School of Public Health, Tokyo, Japan

**Keywords:** Internet addiction, online gaming, prevalence, risk factors, school personnel

## Abstract

**Background:**

The prevalence of Internet addiction (IA) among employed adults has not been reported using a large sample. To clarify the actual status of addictive Internet use among employed adults, this study aimed to evaluate the prevalence and the risk factors of IA and at-risk IA among employed adults in Japan.

**Methods:**

This cross-sectional study surveyed all junior and senior high school personnel in Shimane Prefecture, a rural area in Japan. Eligible participants included 3,211 junior and senior high school personnel (1,259 men and 1,952 women). Participants completed a questionnaire on their activities and factors related to Internet use.

**Results:**

The prevalence of IA and at-risk IA was 0.03% and 4.82%, respectively. Furthermore, game playing was shown to be the Internet activity most closely associated with at-risk IA.

**Conclusions:**

This study showed that around 5% of school personnel in a rural area in Japan are at risk for developing addiction to the Internet and that using the Internet for game playing is related to at-risk IA. Our results suggest that employed adults should be instructed to use the Internet properly.

## INTRODUCTION

Internet addiction (IA) is becoming both a health and social problem among the general population with the spread of Internet access. IA refers to excessive or poorly controlled preoccupations, urges, or behaviors regarding Internet use, which eventually could lead to distress and functional impairment.^1^ IA has been shown to be related to depression, anxiety, aggression, sleep disturbance, and attention deficit hyperactivity disorder (ADHD), and alcohol dependence.^2^^,^^3^

Previous studies have reported that the prevalence of IA among minors is approximately 10% worldwide.^4^^–^^6^ The prevalence of IA among adults has been reported to be around 1.2% to 8%.^7^^–^^14^ However, while the difference in prevalence of IA is reported between employed and unemployed adults,^15^ to our knowledge, no studies have evaluated the prevalence of IA for the employed separately using a large sample.

Although there is no consensus on formal diagnostic criteria for IA, previous studies classified the severity of IA using preliminary cut-off points of the IA scale: non-IA was defined as having complete control over Internet use, at-risk IA was defined as having frequent life problems because of excessive Internet use, and IA was defined as having significant life problems because of excessive Internet use. It has been shown that the individuals with at-risk IA develop more severe health problems than those with non-IA and less severe health problems than those with IA. This suggests that at-risk IA as well as IA individuals have some degree of problematic internet usage.^16^^–^^18^

To clarify the actual status of addictive Internet use among employed adults, this study aimed to evaluate the prevalence and the risk factors of IA and at-risk IA among school personnel in Japan. We used prefecture-wide data obtained from the School Personnel Internet Use Survey of a rural area in Japan.

## METHODS

### Participants and procedures

The survey was a prefecture-wide cross-sectional design. The target population was 4,808 school personnel (ie, school staff including administrators, teachers, and clerks) in all 151 junior and senior high schools in Shimane Prefecture, a rural area in Japan. We sent the study information and questionnaires to all junior and senior high schools in Shimane Prefecture and asked all personnel to participate in the study in the schools whose principals consented to participation. They were informed that the survey was voluntary and that anonymity and confidentiality were ensured. Each school personnel sealed their completed questionnaire in the envelope, and the administrators collected and returned them to us unopened. A completed questionnaire was considered as consent to the study.

Among the 151 schools, 129 schools consented to participate in the survey (response rate: 85.4%). Among the 4,808 school personnel in these schools, 3,305 school personnel responded to the survey (response rate: 68.7%). Ninety-four samples were excluded from the analysis due to missing data. Therefore, data from 3,211 eligible personnel (1,259 men and 1,952 women) were included in the analysis (eligible response rate: 66.8%). This survey was reviewed and approved by the institutional review board of Shimane University (Approved on 7th July, 2015; No. 1863).

### Measures

The questionnaire consisted of two parts. The first part asked participants for biographical and background information, such as sex, age category, position at their school and duration of service for school personnel, average time spent on the Internet per day for the last 1 month (both weekday use and weekend use for work and leisure purposes), the activities engaged in on the Internet for the last 1 month (entertainment, games, communication, shopping), and the devices used for Internet access for the last 1 month (feature phone, smartphone, tablet, desktop computer, laptop computer). The second part of the questionnaire asked participants about IA, which was defined using the Internet Addiction Test (IAT).^19^ The IAT was most commonly used measure in previous studies on IA.^20^ Adequate reliability and validity have been demonstrated for the scale.^21^^,^^22^ The IAT is a self-reported measure comprising 20 items rated on a 5-point Likert scale from “not at all” (1) to “very” (5). The total score of the IAT ranges from 20 to 100. The total score classifies IA as follows: non-IA (total score <40), at-risk IA (total score of 40–59), and IA (total score ≥60).^16^^,^^17^^,^^23^^,^^24^ However, only one respondent had an IAT score of ≥60; therefore, the participants were allocated into either the at-risk IA group (IAT score ≥40) or the non-IA group (IAT score <40).^18^ Acceptable internal consistency was obtained in the sample (Cronbach’s α = 0.88).

### Statistical analysis

We show descriptive statistics of all of the study variables. We compared demographics, time spent on the Internet, activities on the Internet, and devices used for Internet access between the at-risk IA and non-IA groups using the χ^2^ test, Welch *t*-test, or Mann-Whitney U test. A multiple logistic regression analysis was used to assess the contribution of sex, age, activity on the Internet, and device used for Internet access to IA. The explanatory variables theoretically relevant to IA were entered simultaneously into the model. R (version 3.1.1; R Foundation for Statistical Computing, Vienna, Austria)^25^ was used for the analyses. All probability values were two-tailed and at a 5% level of significance.

## RESULTS

Characteristics of the participants between the at-risk IA and Non-IA groups are shown in Table 1. The prevalence of IA in our study was 0.03% (*n* = 1), with 4.82% (*n* = 155) as at-risk IA and 95.14% (*n* = 3,055) as non-IA. Thus, we divided the respondents into two groups: the at-risk IA group and the non-IA group. Mean IAT scores were 45.83 (standard deviation [SD], 6.39) and 24.83 (SD, 4.64) in the at-risk IA and non-IA groups, respectively. Analyses showed that respondents in the at-risk IA group were younger and had a shorter length of service in school than those in the non-IA group. Additionally, the at-risk IA group had a higher proportion of part-time teachers, whereas the non-IA group had a lower proportion of administrators and teachers. The at-risk IA group had a significantly higher portion of owning smartphones and tablets but a lower portion of owning feature phones. There were no group differences related to sex.

**Table 1.** Group characteristics

Variable	Total sample (*n* = 3,211)	At-risk IA (*n* = 156)	Non-IA (*n* = 3,055)	Test statistic	*P* value
IAT score, *Mean* (*SD*)	25.85 (6.54)	45.83 (6.39)	24.83 (4.64)	t(163.41) = 40.37	<0.001
Sex (*n*, %)				χ^2^(1) = 0.91	0.341
Male	1,259 (39.21)	55 (32.26)	1,204 (39.41)		
Female	1,952 (60.79)	101 (64.74)	1,851 (60.59)		
Age, years, *n* (%)				W = 296,798	<0.001
<30	413 (12.86)	48 (30.77)	365 (11.95)		
30–39	571 (17.78)	28 (17.95)	543 (17.77)		
40–49	967 (30.12)	40 (25.64)	927 (30.34)		
50–59	1,098 (34.19)	34 (21.79)	1,064 (34.83)		
>59	162 (5.05)	6 (3.85)	156 (5.11)		
Position at School, *n* (%)				χ^2^(5) = 26.87	<0.001
Administrator	231 (7.19)	5 (3.21)	226 (7.40)		
Teacher	2,142 (66.71)	90 (57.69)	2,052 (67.17)		
Nursing teacher	106 (3.30)	4 (2.56)	102 (3.34)		
Part-time teacher	355 (11.06)	35 (22.44)	320 (10.47)		
Clerk	240 (7.47)	16 (10.26)	224 (7.33)		
Others	137 (4.27)	6 (3.85)	131 (4.29)		
Duration of service, years, *n* (%)				W = 292,561	<0.001
<5	526 (16.44)	53 (34.42)	473 (15.53)		
5–9	361 (11.28)	22 (14.29)	339 (11.13)		
10–19	704 (22.01)	22 (14.29)	682 (22.40)		
20–29	952 (29.76)	40 (25.97)	912 (29.95)		
>29	656 (20.51)	17 (11.04)	639 (20.99)		
Activity on Internet, *n* (% yes)					
Communication	2,648 (82.47)	138 (88.46)	2,510 (82.16)	χ^2^(1) = 3.65	0.056
Gaming	392 (12.21)	47 (30.13)	345 (11.29)	χ^2^(1) = 47.39	<0.001
Shopping	1,784 (55.56)	109 (69.87)	1,675 (54.83)	χ^2^(1) = 13.00	<0.001
Entertainment	2,811 (87.54)	148 (94.87)	2,663 (87.17)	χ^2^(1) = 7.39	0.007
Device for Internet access, *n* (% yes)					
Feature phone	1,145 (35.66)	43 (27.56)	1,102 (36.07)	χ^2^(1) = 4.32	0.038
Smartphone	2,012 (62.66)	113 (72.44)	1,899 (62.16)	χ^2^(1) = 6.27	0.012
Tablet	917 (28.56)	58 (37.18)	859 (28.12)	χ^2^(1) = 5.54	0.019
Desktop computer	1,076 (33.51)	60 (38.46)	1,016 (33.26)	χ^2^(1) = 1.58	0.209
Laptop computer	2,512 (78.23)	130 (83.33)	2,382 (77.97)	χ^2^(1) = 2.20	0.138
Number of devices, *Mean* (*SD*)	2.38 (0.82)	2.59 (0.87)	2.36 (0.82)	t(169.19) = 3.00	0.003

IA, Internet addiction; IAT, Internet Addiction Test; SD, standard deviation.

Mann-Whitney U tests showed that the at-risk IA group spent a longer time on the Internet regardless of the day of week or purpose of use than the non-IA group (weekday use for leisure: U = 107,179.5, *P* < 0.001; weekday use for work: U = 167,837.5, *P* < 0.001, weekend use for leisure: U = 92,205, *P* < 0.001; and weekend use for work: U = 173,290.5, *P* < 0.001, Table 2).

**Table 2.** Time spent on the Internet access between the at-risk Internet addiction (IA) and non-IA groups

Time spent on Internet access	Total sample (*n* = 3,211)	At-risk IA (*n* = 156)	Non-IA (*n* = 3,055)
Weekday use for leisure, hours, *n* (%)			
0	208 (6.50)	3 (1.92)	205 (6.73)
0–0.5	1,185 (37.02)	11 (7.05)	1,174 (38.56)
0.5–1	821 (25.65)	29 (18.59)	792 (26.01)
1–2	780 (24.37)	65 (41.67)	715 (23.48)
2–3	160 (5.00)	31 (19.87)	129 (4.24)
3–4	27 (0.84)	10 (6.41)	17 (0.56)
4–5	13 (0.41)	2 (1.28)	11 (0.36)
>5	7 (0.22)	5 (3.21)	2 (0.07)
Weekday use for work, hours, *n* (%)			
0	179 (5.59)	4 (2.56)	175 (5.77)
0–0.5	1,292 (40.36)	36 (23.08)	1,256 (41.25)
0.5–1	734 (22.93)	34 (21.79)	700 (22.99)
1–2	690 (21.56)	51 (32.69)	639 (20.99)
2–3	164 (5.12)	16 (10.26)	148 (4.86)
3–4	60 (1.87)	6 (3.85)	54 (1.77)
4–5	28 (0.87)	2 (1.28)	26 (0.85)
>5	40 (1.25)	7 (4.49)	33 (1.08)
Weekend use for leisure, hours, *n* (%)			
0	189 (5.90)	1 (0.64)	188 (6.17)
0–0.5	850 (26.55)	5 (3.21)	845 (27.75)
0.5–1	736 (22.99)	16 (10.26)	720 (23.65)
1–2	898 (28.05)	47 (30.13)	851 (27.95)
2–3	311 (9.72)	37 (23.72)	274 (9.00)
3–4	100 (3.12)	17 (10.90)	83 (2.73)
4–5	47 (1.47)	20 (12.82)	27 (0.89)
>5	35 (1.09)	13 (8.33)	22 (0.72)
Weekend use for work, hours, *n* (%)			
0	1,297 (40.52)	43 (27.56)	1,254 (41.18)
0–0.5	1,083 (33.83)	40 (25.64)	1,043 (34.25)
0.5–1	388 (12.12)	20 (12.82)	368 (12.09)
1–2	346 (10.81)	28 (17.95)	318 (10.44)
2–3	60 (1.87)	21 (13.46)	39 (1.28)
3–4	16 (0.50)	1 (0.64)	15 (0.49)
4–5	6 (0.19)	1 (0.64)	5 (0.16)
>5	5 (0.16)	1 (0.64)	4 (0.13)

IA, Internet addiction.

Results of a multivariable logistic regression analysis are shown in Table 3. Age and game playing were the factors that significantly distinguished at-risk IA from non-IA (age <30: adjusted odds ratio 2.69; game playing: adjusted odds ratio 2.50). Variance inflation factors for the explanatory variables ranging from 1.03 to 4.63 support the absence of serious multicollinearity.

**Table 3.** Odds ratios and 95% confidence intervals to at-risk Internet addiction from a logistic regression analysis

Variable	Crude Odds Ratio (95% CI)	Odds ratio (95% CI)	*P* value
Sex			
Male	Reference	Reference	—
Female	1.19 (0.85–1.67)	1.28 (0.90–1.81)	0.171
Age, years			
<30	4.12 (2.61–6.49)	2.69 (1.69–4.27)	<0.001
30–39	1.61 (0.97–2.69)	1.16 (0.70–1.92)	0.573
40–49	Reference	Reference	—
50–59	0.35 (0.85–2.15)	0.78 (0.48–1.27)	0.318
>59	1.20 (0.50–2.91)	1.15 (0.46–2.83)	0.766
Activity on Internet			
Communication	1.66 (1.01–2.74)	1.22 (0.72–2.07)	0.465
Gaming	3.39 (2.36–4.85)	2.50 (1.70–3.66)	<0.001
Shopping	1.91 (1.35–2.71)	1.40 (0.96–2.04)	0.083
Entertainment	2.72 (1.33–5.59)	1.83 (0.87–3.84)	0.110
Device for Internet access			
Feature phone	0.67 (0.47–0.97)	0.92 (0.44–1.96)	0.837
Smartphone	1.60 (1.12–2.29)	0.84 (0.39–1.84)	0.669
Tablet	1.51 (1.08–2.11)	1.19 (0.83–1.69)	0.338
Desktop computer	1.25 (0.90–1.75)	1.32 (0.92–1.89)	0.130
Laptop computer	1.41 (0.92–2.17)	1.42 (0.90–2.25)	0.133

CI, confidence interval.

## DISCUSSION

Our study showed that around 5% of junior and senior high school personnel in Japan showed at-risk IA, whereas few had IA, based on a large sample with a high response rate. Furthermore, the results showed that at-risk IA was strongly associated with both age under 30 and gaming on the Internet. This study clarified the prevalence of IA and at-risk IA and the risk factors among employed adults in Japan. In Asian countries, despite there being many studies of minors, relatively few studies on IA have been conducted among adults using a large sample.^13^^,^^14^ Thus, this study is important for finding further results on IA among adults in an Asian country.

The results obtained indicated that there was a lower prevalence of IA in comparison with that in previous studies.^8^^,^^11^^,^^13^ This low prevalence observed in our study may be partly attributable to the inclusion of only employees. Working may prevent them from using the Internet limitlessly and inhibit the progression of IA. In contrast, the present prevalence of at-risk IA is almost equivalent to that reported in the Western countries. The results suggest smaller differences in the prevalence of at-risk IA between employed and non-employed adults.

We found at-risk IA to be associated with both age under 30 and gaming on the Internet. Our results on the association between younger age and at-risk IA are similar to previously reported results in adults.^8^^,^^12^^,^^26^^,^^27^ This may be because the younger age group is more accustomed to using the Internet due to exposure to the Internet environment from early childhood. Furthermore, school administrators and teachers are likely to show non-IA, whereas part-time teachers are likely to show at-risk IA. This may be attributable to the older age of the administrators and teachers and because part-time teachers are younger and work fewer hours. However, no association was found between sex and at-risk IA. Although previous studies have reported that men were more likely to exhibit IA and at-risk IA in minors, a previous study has suggested that a relationship between IA and gender difference does not exist in adults.^13^^,^^28^ Thus, we could find no gender difference due to the inclusion of only adult participants in this study.

Similar to previous studies on the association between IA and online gaming in adults, our results also indicated that online gaming is one of the risk factors for at-risk IA rather than other activities on the Internet. It is reported that online gaming is more strongly related to IA in comparison with other activities on the Internet.^29^^,^^30^ In addition, the Diagnostic and Statistical Manual of Mental Disorders 5th ed. (DSM-V)^31^ refers specifically to the addiction to online games (ie, Internet Gaming Disorders). IA caused by gaming binges may result not only from attraction with the game itself but also the bidirectional communication occurring during online gaming. Some online games enable game play with other online game players (eg, massively multiplayer online role-playing games). These games may provide opportunities to communicate with other players and receive admiration for contributions to a game task and also make it difficult to play online games at one’s own pace. Thus, it could have a negative effect on a person’s ability to control online gaming.

Adults need to be provided with intervention for the prevention and treatment for IA and at-risk IA. Excessive Internet use can cause sleep disorders, general fatigue, and mental problems. It may reduce both mental and physical health among school personnel with IA. Our results suggest that adults should be provided with information and mental health services to use the Internet properly to improve their mental health.

A similar study investigated the prevalence of IA in Japan in a large cohort of junior and senior school students.^32^ The study reported a higher prevalence of IA in comparison with the results of the present study and sex differences in prevalence, which may be because of differences in age of the participant between the studies. Regarding the risk factors for IA, online gaming was associated with IA in both studies. In contrast, in their study social-networking services were associated with IA in female students. In the present study, the participants were adults, and that may partly explain the lack of the relationship between communication on the Internet and IA.

This study has several limitations. First, our study uses a cross-sectional design, which does not prove a causal relationship. Second, since there is no consensus on the formal diagnostic criteria and gold standard measures for IA, the diagnostic accuracy properties of the IAT cut-off scores are yet to be established. Third, the participants are limited to school personnel in a rural area in Japan. Thus, the results cannot be generalized to populations with different backgrounds. Furthermore, school personnel may not be representative of all of the employed population, even though the sample included administrators, clerks, and other personnel who work in the schools, as well as teachers.

In conclusion, this study showed that around 5% of school personnel in a rural area in Japan are at risk for developing addiction to the Internet. At-risk IA was found to be associated with the use of the Internet for game playing. Our results suggest that employed adults should be instructed to use the Internet properly.
